# The effect of follicular and ampullary fluid extracellular vesicles on bovine oocyte competence and *in vitro* fertilization rates

**DOI:** 10.1371/journal.pone.0325268

**Published:** 2025-06-06

**Authors:** Zohreh Pakniyat, Mehdi Azari, Mojtaba Kafi, Mehran Ghaemi, Seyed Mohammad Amin Hashemipour, Amin Safaie, Davoud Eshghi

**Affiliations:** 1 Department of Clinical Sciences, School of Veterinary Medicine, Shiraz University, Shiraz, Iran; 2 Department of Pathobiology, Faculty of Veterinary Medicine, Ferdowsi University of Mashhad, Mashhad, Iran; 3 Department of Physics, Faculty of Science, Shiraz University, Shiraz, Iran; Shiraz University of Medical Sciences, IRAN, ISLAMIC REPUBLIC OF

## Abstract

Follicular fluid from preovulatory follicles as well as ampullary fluid from slaughtered cows at the early metestrus were collected for isolation of EVs. Excellent and good quality bovine oocytes were selected and distributed into four groups: control group which did not receive EVs, the FFEV group which were exposed to 40 μg/ml of follicular fluid EVs for the first 18 hours of the culture, the FFAFEV group which received 40 μg/ml of follicular fluid EVs for the first 18 hours of the culture, followed by 3.4 μg/ml of ampullary fluid EVs for the remaining 4.5 hours, and the AFEV group which were exposed to 3.4 μg/ml of ampullary fluid EVs for the final 4.5 hours of the culture. After a total incubation period of 22.5 hours, the COCs were evaluated for nuclear maturation, expression of some relevant genes, and Raman spectra from different areas of the representative matured oocytes (Experiment 1). In addition, fertilization rate of the oocytes was assessed after addition of EVs to the maturation medium (Experiment 2). The maturation and fertilization rates, as well as the expression of *TNFAIP6*, *HAS2*, and *GDF9* genes, were significantly higher in the EVs treatment groups compared to the control group (p ≤ 0.05). Furthermore, the Raman microspectroscopy revealed a higher number of mitochondria (1602 cm^-1^), increased levels of unsaturated lipids (1655 cm^-1^) and a favorable phenylalanine to carbohydrate ratio (1002/1037, serving as a marker for oocyte quality, in the FFAFEV group compared to the control group. Additionally, there was a lower concentration of saturated lipids (2883 cm^-1^) in the FFAFEV group than in the control group. In conclusion, our findings showed that supplementation of the oocyte maturation media with follicular and ampullary fluid EVs positively influenced oocyte quality and enhanced *in vitro* maturation, fertilization rates, and the relevant gene expression.

## Introduction

Assisted reproductive technologies (ART) provide solutions to treat poor fertility and even sterility in humans and animals. *In vitro* production of embryos is a well-accepted and routine method to cure reproductive failure in fertility clinics around the world. Despite many advances in understanding the physiology of reproduction and innovative ART methods however, the rate of *in vitro* competent embryo production remains lower than *in vivo* embryos. The primary reason is that the *in vitro* environment fails to perfectly replicate the conditions found *in vivo* [1]. Therefore, providing a more appropriate *in vitro* environment will aid to improve developmental competence of produced *in vitro* embryos [2]. Various approaches have been used to enhance the quality of the *in vitro* environment for production of higher competent embryos including using the oviduct cells [3], oviduct fluid [4], follicular fluid [5], and more recently addition of Extracellular vesicles (EVs) originated from different segments of the reproductive system [6].

EVs, a new medium for cellular communication, contribute to the maturation of oocytes and early embryo development *in vivo* [7]. They function via changes in gene expression in different parts of the reproductive system including ovarian follicular cells, oviductal and the uterine cells [8]. There are three major types of EVs: exosomes, microvesicles, and apoptotic bodies. The diameter of exosomes, microvesicles and apoptotic bodies is ranging from 30–150 nm, 100–1000 nm, and 1–5 µm, respectively. Exosomes are produced by the fusion of multivesicular endosomes with the plasma membrane, while microvesicles are released from the plasma membrane [9]. These vesicles exchange biological materials such as miRNAs, mRNAs, proteins, and lipids [10]. Studies have demonstrated that EVs are secreted by various sources including blood, urine, follicular fluid [7], oviduct [11], uterus [12], and embryo [13]. Oviductal EVs play crucial roles in regulating gamete function, fertilization, and embryo development. Therefore, incorporating oviductal fluid EVs into *in vitro* culture media could help to mimic the natural oviduct environment, leading to improved embryo quality and reduced polyspermy [10]. In addition, Oviduct epithelial cell-derived EVs decrease apoptosis by reducing reactive oxygen species [14]. Moreover, follicular fluid EVs have an important role in mediating the communication between the oocyte and the surrounding somatic cells by transporting bioactive molecules to promote follicular growth and oocyte maturation [15]. These types of EVs can protect oocytes from stress [16]. They also improve the cumulus expansion and increase embryo quality [5]. Thus, supplementing follicular and ampullary EVs into *in vitro* maturation media has a positive impact on embryo development [17] and can optimize outcomes across various assisted reproductive technologies.

The quality of the oocyte is widely recognized as a crucial factor in determining the potential normal development of embryos. Morphological evaluation is the most widely used procedure to examine the oocyte competence in the laboratory. Investigating gene expression during oocyte and embryo culture is however a more accurate way to study the oocyte competence for the research purposes. Growth differentiation factor 9 (*GDF9*) is secreted from the oocyte, necessary for cumulus cell expansion, oocyte development, and preventing cumulus cell apoptosis [18]. Hyaluronic acid synthase 2 (*HAS2*) and Tumor necrosis factor, a-induced protein 6 (*TNFAIP6*) are required for the formation of the extracellular matrix during cumulus cell expansion, and their expression is directly linked to oocyte development [19]. In addition, BCL2-associated X protein (*BAX*) is involved in regulating apoptosis and is used to analyze apoptosis in oocytes and embryos [20]. There is a growing interest in developing non-invasive methods for assessing oocyte quality to improve the outcomes of assisted reproductive technologies in both medical and veterinary fields. Conventional methods typically rely on visual assessment of oocyte morphology, but these lack the biochemical detail needed to accurately predict developmental outcomes [21]. Raman microspectroscopy (RMS) offers a promising non-invasive approach, providing high-resolution, label-free molecular analysis of oocytes [22]. RMS works by detecting unique vibrational signatures generated when laser light interacts with molecular bonds, enabling precise identification of cellular components, including proteins, lipids, carbohydrates, and nucleic acids within oocytes [23]. The physical basis of RMS is based on inelastic scattering, in which incident photons interact with molecular vibrations, leading to a slight energy shift that reveals specific vibrational modes unique to each molecule. These shifts, known as Raman shifts, are represented in a spectrum that provides a “molecular fingerprint” of the sample [22,24]. Through this method, subtle biochemical variations associated with both normal and abnormal conditions can be identified reflecting oocyte quality. Recent research has shown that RMS is capable of differentiating oocytes by analyzing their lipid and protein compositions, identifying biochemical alterations throughout various developmental phases, and evaluating the impact of cryopreservation on cellular integrity and viability [25,26]. Importantly, the microscopic mapping capabilities of RMS enable researchers to visualize spatial biochemical differences within the oocyte, facilitating real-time monitoring of key biomolecules during oocyte maturation and early embryo development [27]. This non-destructive approach offers significant advantages over traditional methods, positioning RMS as a valuable tool for advancing ART by providing detailed molecular insights into oocyte health and viability.

Previous studies have focused on the effect of EVs on *in vitro* embryo development [17] however, the specific effects of concomitant addition of bovine follicular and ampullary oviductal fluid EVs to the *in vitro* maturation (IVM) medium have not been examined on the oocyte quality. In addition, there is no study available using RMS to examine the quality of the oocyte matured *in vitro* in the bovine. Therefore, the present study was designed to examine the effect of follicular and ampullary oviductal fluid EVs on *in vitro* maturation and fertilization of bovine oocytes, and further to examine the expression of relevant genes to oocyte maturation. In addition, the composition of the *in vitro* matured oocytes was evaluated using RMS.

## Materials and methods

### Ethical considerations

This research received approval from the animal research committee of the School of the Veterinary Medicine, Shiraz University (1GCB2M370068).

### Bovine follicular and ampullary oviductal fluid collection

Follicular fluid (FF) was collected from 10 Holstein virgin heifers in 14–16 months. Animals were synchronized by two intramuscular administrations of prostaglandin F2α (500 μg Cloprostenol sodium; Estroplan Parnell Living Science, Alexandria, NSW, Australia), 11 days apart. The follicular fluid was aspirated from preovulatory follicles (10–15 mm) utilizing a long fine needle in conjunction with caudal epidural anesthesia (2% Lidocaine hydrochloride; Pasteur Institute, Iran, 0.2 mg/kg) as previously described by Kafi et al. (2017) [28]. Oviducts and ovaries from slaughtered cows were collected at the early postovulatory phase of the estrous cycle based on the presence of a corpus hemorrhagicum. The oviducts and ovaries were delivered to the laboratory in ice-pack containers within two hours after collection. The connective tissue encasing the oviducts was excised using a scalpel blade, followed by two washes of the oviducts in a 0.9% sodium chloride solution and a 70% ethanol solution. The oviducts were trimmed, washed with PBS and then the ampulla was detached at the junction between the ampulla and isthmus, a point characterized by a significant decrease in the diameter of the oviduct [11]. Next, the lumen of the ampulla was flushed gently with 1 mL sterile PBS using a 22 G catheter. The ampullary Fluid (AF) samples were then stored at −80°C until further analysis.

### Follicular and ampullary oviductal fluid extracellular vesicles isolation

Follicular and ampullary fluid EVs were isolated using an Exocib kit (Cib Biotech Company, Iran). The Exocib kit provides a rapid method for separating and purifying EVs from biological fluid. This kit contains two reagents, A and B. Reagent A was vortexed and heated to 37°C until crystals disappear. Samples of FF and AF were thawed at 4°C and spun at 3000 rpm for 10 minutes to remove particles and debris. Next, the samples were filtered through a 0.22 µm filter. Reagent A was added in a 5:1 ratio (5 parts sample to 1 part reagent A), and the tubes were vortexed for 5 minutes until thoroughly mixed. Then, the mixture was incubated overnight for 12 hours at 4°C, and the tubes were shaken every hour according to the kit manufacturer’s recommendation. After 12 hours, the tubes were vortexed for 1 minute to mix thoroughly and spun for 40 min at 3000 rpm at 4°C, and the supernatant was entirely removed and discarded. Finally, the EVs pellet was re-suspended with 100 microliters of reagent B, and these solutions were stored at −80°C until use.

The protein concentration of follicular and ampullary fluid EVs was determined by the Bradford test (ProtoCib kit, Cib Biotech company, Iran) according to the manufacturer’s instructions. Protein concentration in the FF EVs and AF fluid EVs were 20 mg/ml and 0.3 mg/ml, respectively.

### Characterization of extracellular vesicles

#### Transmission Electron Microscopy (TEM).

**Fig 1:**
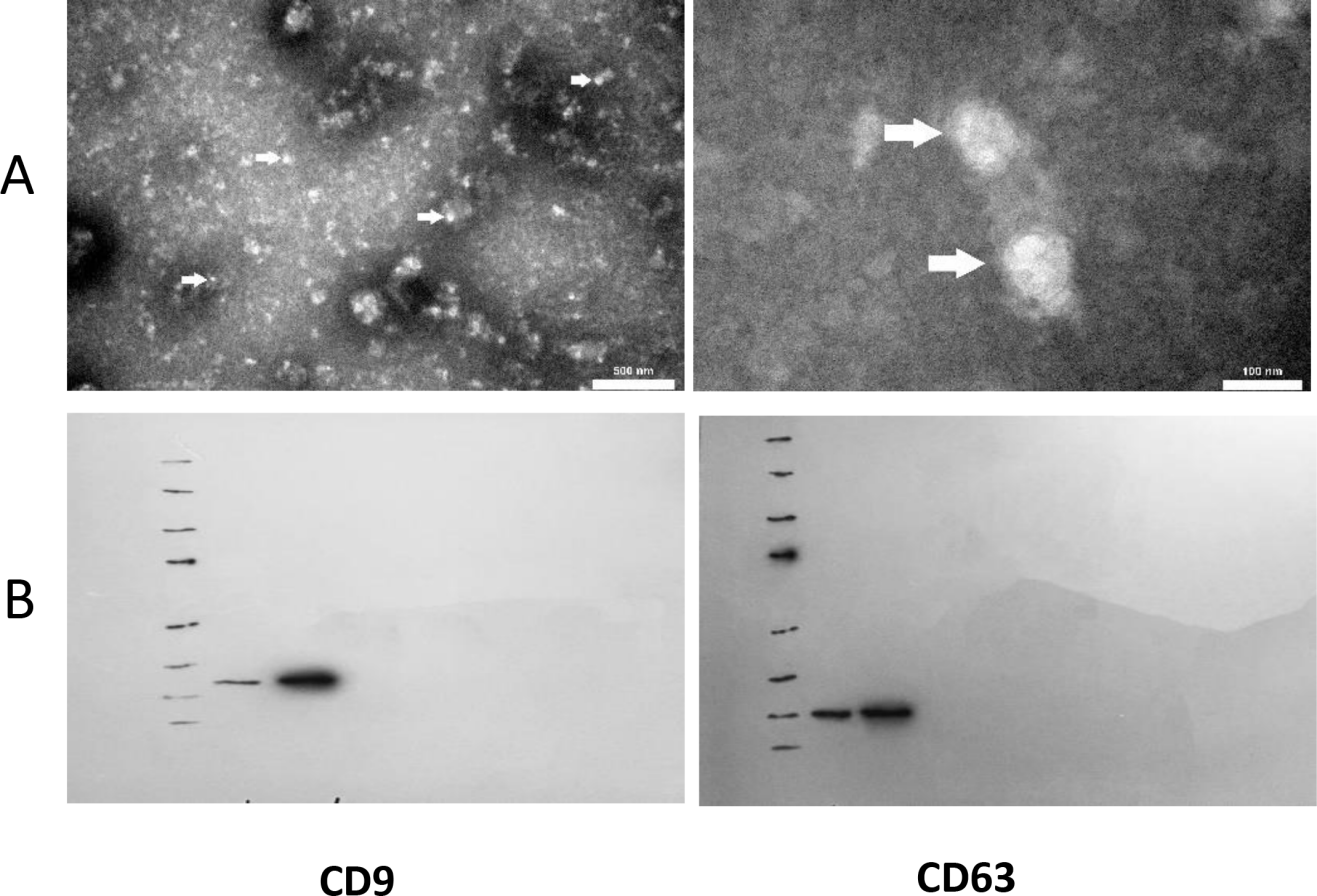
(A) Transmission electron microscopy showing morphological structures of EVs (white arrows) from AF and FF, with sizes ranging from 100 nm to 400 nm in diameter (scale bar: 500 nm and 100 nm). (B) Western blotting analysis showed the presence of EV-associated proteins CD9 (25 kDa), CD63 (27 kDa) in AF and FF.

Extracellular vesicles derived from FF and AF were examined using transmission electron microscopy (TEM). In summary, the EVs were fixed overnight in a solution containing 2% glutaraldehyde and 0.1 M cacodylate buffer, followed by washing with 0.1 M sodium cacodylate buffer. Each sample was deposited on precoated formvar/carbon support film copper mesh electron microscopy grids. Each grid underwent rinsing and filtration using double-distilled water, followed by a staining process with 1% uranyl acetate for 45 seconds [17]. All samples were examined using electron microscopy (EM 208S, Philips, Netherlands) (Fig 1A).

#### Western blotting.

Western blotting (WB) was conducted using EV-specific markers (CD63 and CD9) to assess the presence of EVs and other nanoparticles in all samples. The lysates were separated through centrifugation at 14,000 rpm for 20 minutes at 4°C. The concentration of proteins of the lysates was assessed using the BCA Protein Quantification kit, following the guidelines provided by the manufacturer. The lysates were combined with an equivalent volume of 2X Laemmli sample buffer. The lysates (15 μg) were then subjected to SDS-PAGE after a 5 minutes boiling and subsequently transferred to a 0.2 μm immune-Blot™ polyvinylidene difluoride (PVDF) membrane (Cat No: 162–017777; Bio-Rad Laboratories, CA, USA). The membranes were subsequently blocked with 5% BSA (Cat No: A-7888; Sigma Aldrich, MO, USA) in 0.1% Tween 20 for 1 hour. Then, the membranes were incubated with Anti-CD9 (Cat No: ab307086, Abcam), and Anti-CD63 (Cat No: ab231975, Abcam), for 1 hour at room temperature. The membranes were then subjected to three washes with TBST and subsequently incubated with goat anti-rabbit IgG H&L (HRP) (Cat No: ab6721; Abcam) secondary antibodies. Finally, the membranes were incubated with enhanced chemiluminescence (ECL) for 1–2 minutes (Fig 1B) [29].

#### Study design.

To examine the effect of EVs on the *in vitro* oocyte nuclear maturation and fertilization, Excellent and good quality COCs were distributed into four treatment groups after collection.

Group 1) COCs were cultured in standard IVM medium with no EVs for 22.5 hours (Control group),

Group 2) COCs were incubated in IVM medium enriched with 40 μg/ml of FF EVs for 18 hours. Following this period, the IVM medium was substituted with fresh, equilibrated IVM medium for an additional 4.5 hours (FFEV group),

Group 3) COCs were cultured in IVM medium supplemented with 40 μg/ml FF EVs for 18 hours. After 18 h, the IVM medium was refreshed with fresh equilibrated IVM medium supplemented with 3.4 μg/ml AF EVs for the remaining 4.5 hours (FFAFEV group),

Group 4) COCs were cultured in standard IVM media without EVs for 18 hours. After that, the IVM medium was replaced with fresh equilibrated IVM medium supplemented with 3.4 μg/ml AF EVs for the remaining 4.5 hours (AFEV group).

First, several different concentrations of FF and AF EVs (first 18 hours of culture) and ampullary oviduct fluid EVs (last 4.5 hours of culture) were used to determine the optimal concentration. The study’s optimal concentration was determined by the oocyte cleavage rate after *in vitro* fertilization. For FFEVs, 40 μg/ml and AFEVs, 3.4 μg/ml were obtained. All cultural conditions were uniformly applied across the four experimental groups. The experiments was carried out in five separate replicates.

### Experiment 1

#### Effect of EVs on *in vitro* oocyte nuclear maturation.

Cattle ovaries were collected from the Shiraz abattoir and subsequently transported to the laboratory within 2 hours in a physiological saline solution of 0.9% NaCl, while being kept at a temperature between 32 and 35°C. In the laboratory, the ovaries underwent a sequential washing process using a 0.9% NaCl solution. Subsequently, follicles ranging in diameter from 3 to 8 mm were aspirated utilizing an 18-gauge needle connected to a 10 mL syringe. The collected FF was transferred to a conical tube and subsequently incubated in a water bath maintained at 37°C for 15 minutes. Following this, the pellet along with 5 mL of the FF was transferred to a 90 mm petri dish. Oocytes exhibiting intact compact layers of cumulus cells and a uniform cytoplasm were then selected for IVM [28].

Selected COCs were cultured in a maturation medium consisting of TCM-199 (Sigma, UK) supplemented with 0.1 IU/mL FSH (Follitropin alfa 75 IU, Cinna Gene company, Iran), 5 IU/mL hCG (Folignan, Daroupakhsh, Iran), 50 μg/mL gentamicin and 10% FCS. A total of 30–50 COCs were incubated in 500 μL equilibrated maturation medium in four-well culture dishes in each group in each replicate (SPL, Korea) for 22.5 hours at 39°C in 5% CO_2_ under maximum relative humidity. After maturation, cumulus cells were mechanically denuded by sequential pipetting. Then the oocytes were mounted on a slide, covered with a cover slip, and fixed in acetic alcohol (1:3) solution for at least 24 hours. The nuclear morphology evaluation was carried out on the oocytes by staining them with 1% aceto-orcein (1% orcein in 45% glacial acetic acid) and examining them with a light microscope at ×400 magnifications. Oocytes were classified as follows: immature (did not reach metaphase), mature (presented a metaphase II plate and 1^st^ polar body), metaphase І, abnormal (any chromosomal aberrations such as diploid, abnormal metaphase II, multidirectional spindle and chromosomal dispersion), and degenerate (presented diffuse or undefined chromatin) [30].

#### Effect of EVs on the gene expression in the matured oocytes.

***Sample collection, RNA extraction and cDNA synthesis*:** After maturation, 40–50 expanded COCs were collected from each experimental group with 100 μL IVM media and stored at −70°C until RNA extraction. According to the manufacturer’s instructions, total RNA was isolated using a RNeasy Micro Kit (DenaZist Asia, Iran). The concentration of extracted RNA was determined by NanoDrop spectrophotometer (Thermo Fisher Scientific™, and using the absorbance value of 260 nm). Based on the manufacturer’s instructions, reverse-transcription reactions were performed using an EasyTM cDNA Synthesis Kit (Parstous, Mashhad, Iran) and random hexamer as a primer.

***Real-time polymerase chain reaction*:** The relative quantitative real-time PCR reactions to assess the mRNA expression of the candidate genes (*GDF9*, *HAS2*, *TNFAIP6*, *BAX*) and housekeeping gene (*GAPDH*) were performed in a real-time PCR system (LightCycler® 96, I Roche Life Science, Germany). Primers were designed using Beacon Designer software (Premier Biosoft). Table 1 lists the primer pair sequences and other information for each gene. The real-time PCR mix in each well included the following: 7 μL SYBR®Green PCR Master Mix (Ampliqon, Denmark), 0.1 μL of each forward and reverse primers (10 μM), 4.3 μL nuclease-free water and 2.5 μL of cDNA (12.5 ng/μL) in a final volume of 14 μL. Then the cyclic condition consisted of a preincubation step of 95°C for 5 minutes, followed by a three-step amplification in 45 cycles: denaturation at 95°C for 10 s, annealing (Table 1) for 20 s, and extension at 72°C for 10 s. The expression of each gene was normalized against the housekeeping gene (*GAPDH*) (internal control). The Ct data were transferred to Microsoft Excel for analysis. A comparison of relative gene expression between calibrator and treatment groups was made using the ΔΔCT method. 2^^^ΔΔCT values (fold change) were calculated for each gene in all experimental groups.

**Table 1:** The primers used in the quantitative real-time PCR reactions.

Gene name	Forward	Reverse	cDNA product size	Tm (Annealing)
*GAPDH*	5`-GCTCTCTGCTCCTGCC-3`	5`-CCGTTCTCTGCCTTGACTG-3`	259	59°C
*GDF9*	5`-CCAGGCAGCAGGAACC-3`	5`-CACCAGAGGCTCAAGAGG-3`	240	60°C
*HAS2*	5`-GGATGAGTCGCACAAAGA-3`	5`-TTAAATCTGGACATCTCCCC-3`	256	59°C
*BAX* *TNFAIP6*	5`-CCCGAGAGGTCTTTTTCC-3` 5`-GCTCACGGATGGGGAT-3`	5`-CGTCCCAACCACCCTG-3` 5`-GCTTGTATTTGCCAGACC-3`	216 100	60°C 60°C

#### Raman microspectroscopy.

Oocytes were cultured as previously mentioned in Experiment 1. After 22.5 hours, morphologically matured oocytes in each group were fixed in 2% w/v paraformaldehyde (PF) and examined with RMS. Briefly, washing medium (PBS/BSA) was placed at 39°C and 5% CO_2_ for at least 1 hour, while the 2% PF was kept at room temperature. Oocytes were washed in warm PBS/BSA, and the cumulus cells were mechanically denuded by sequential pipetting. Then, oocytes were transferred from the washing media to 2% PF for 20 minutes at room temperature. Finally, oocytes were transferred to the 4-well dishes filled with 500 µl of PBS/BSA, and then refrigerated until examination [31].

In this study, Raman spectra were acquired using a HORIBA LabRAM HR Evolution Raman spectrometer (HORIBA Scientific Ltd.), integrated with a confocal BXFM microscope and a Syncerity OE CCD detector, maintained at −60°C for optimal performance. To ensure high accuracy and reliability of measurements, a rigorous calibration process was conducted before data collection. This involved analyzing the Raman spectrum of a pure silicon wafer, enabling precise alignment of the spectrometer to the 520.7 cm ⁻ ¹ silicon reference peak. This calibration step was critical for maximizing the accuracy and consistency of the spectral data throughout the experiment. Raman spectra were obtained using a 532 nm excitation laser operating at 100 mW power. A × 50 objective lens was utilized to enhance measurement precision, while the confocal microscope’s pinhole was set to 200 micrometers. This setup minimized fluorescence interference and precisely localized the sampling area for improved spectral clarity. Spectra for oocyte samples across all four experimental groups were collected within the wavenumber range of 500–3000 cm ⁻ ¹. This range encompasses the fingerprint region, capturing critical biochemical information relevant to biological samples. Each spectrum acquisition lasted 15 seconds, with data averaged from five individual measurements per sample to ensure reliability. The total number of collected spectra per group was the same as the number of oocytes used for spectral collection in each group (n = 13).

### Experiment 2

#### Effect of EVs on *in vitro* oocyte fertilization.

To investigate the effect of EVs on bovine oocyte *in vitro* fertilization, maturation of the COCs was performed as previously mentioned in Experiment 1. Then, the matured COCs were transferred to the fertilization medium (modified Tyrode’s medium) for *in vitro* fertilization. Frozen-thawed commercial semen with proved fertility was used in the current experiment.. After 21 hours, the presumptive zygotes were denuded and placed on a slide, covered with a cover slip, and fixed in acetic alcohol (1:3) solution for at least 24 hours. The fertilization rate was evaluated on the presumptive zygotes by staining them with 1% aceto-orcein (1% orcein in 45% glacial acetic acid) and examining the zygotes with a light microscope at ×100 and ×400 magnifications. Presumptive zygotes were categorized as fertilized (presence of two pronuclei), unfertilized ova (without pronuclear conformation and no existence of sperm in the ooplasm and nonappearance of the second polar body), and polyspermy (creation of more than two pronuclei) based on their status [28].

#### Effects of EVs on *in vitro* fertilization rate following Intra Cytoplasmic Sperm Injection (ICSI).

The ICSI procedure was performed according to the standard method previously described by Bevacqua et al. (2010). Briefly, after *in vitro* maturation, cumulus cells were removed, and only oocytes with a visible polar body were used for ICSI. Swim-up prepared, morphologically normal sperm were selected, immobilized, aspirated, and then were injected into an oocyte. Then, the injected oocytes were chemically activated using calcium ionophore A23187 and ethanol 7% [32].

To assess the fertilization rate, presumptive zygotes were transferred on a slide after a 15-hour incubation period, then stained with 1% aceto-orcein (1% orcein in 45% glacial acetic acid), and after 24 hours examined for observing polar bodies and pronuclei with a light microscope at ×100 and ×400 magnifications.

### Statistical analyses

The data were analyzed using SPSS statistical software (version 26.0; SPSS for Windows; SPSS Inc., Chicago, Illinois, USA). The Kolmogorov-Smirnov test was used to assess normality and homogeneity of variance. Comparisons of the oocyte nuclear maturation, fertilization and mRNA transcript expression among groups were made using one-way analysis of variance (ANOVA) followed by Tuckey’s post hoc test. Data were expressed as mean ± standard error. The data obtained from Raman microscopy were subjected to Principal Component Analysis (PCA) and PCA-Linear Discriminant Analysis (PCA-LDA) in MATLAB. In detail, to enhance the spectral data quality, preprocessing techniques were performed in MATLAB (version R2024a, MathWorks Inc, MA, USA). These included polynomial baseline correction to reduce fluorescence interference, vector normalization for noise elimination, and Savitzky-Golay smoothing to refine signal clarity. Additionally, average processing of the Raman spectra was conducted to ensure consistency and accuracy across all samples. This statistical analysis enabled the detection of spectral variations and provided insight into the effects of FFAFEV treatment on the samples, based on their distinct molecular signatures. Values ≤ 0.05 were considered statistically significant.

## Results

### Experiment 1

#### Effect of EVs on *in vitro* oocyte nuclear maturation rate.

The results of *in vitro* maturation are presented in Table 2. The nuclear maturation rate was significantly higher in the FFAFEV and FFEV groups compared to the control and AFEV groups (p ≤ 0.05). There was no significant differences in the oocyte maturation rate between the FFAFEV and FFEV groups and further no difference was observed in the oocyte maturation rate between the control and AFEV groups (p > 0.05). Additionally, the immature oocyte in the FFAFEV group was significantly lower than the other groups (p ≤ 0.05).

**Table 2:** Effect of addition of EVs on oocyte nuclear maturation (mean ± standard error).

Treatment	N	Mature n (%)	M1 n (%)	Immature n (%)	Degenerate n (%)
Control	149	112 (74.65 ± 1.80)^a^	19 (13.64 ± 2.76)	12 (7.85 ± 1.91)^a^	6 (3.84 ± 1.31)
FFEV	165	132 (80.18 ± 0.61)^b^	15 (9.04 ± 0.71)	11 (7.23 ± 1.12)^a^	7 (3.54 ± 1.18)
FFAFEV	163	139 (85.00 ± 1.38)^b^	14 (8.87 ± 1.16)	2 (1.18 ± 0.76)^b^	8 (4.94 ± 1.12)
AFEV	170	120 (71.47 ± 1.52)^a^	17 (8.56 ± 1.93)	13 (8.74 ± 2.07)^a^	20 (11.21 ± 1.25)

FFEV: cultured with Follicular fluid EVs; FFAFEV: cultured with Follicular fluid EVs and Ampulla fluid EVs; AFEV: cultured with Ampulla fluid EVs.

#### Effect of EVs on gene expression in the *in vitro* matured oocytes.

**Fig 2:**
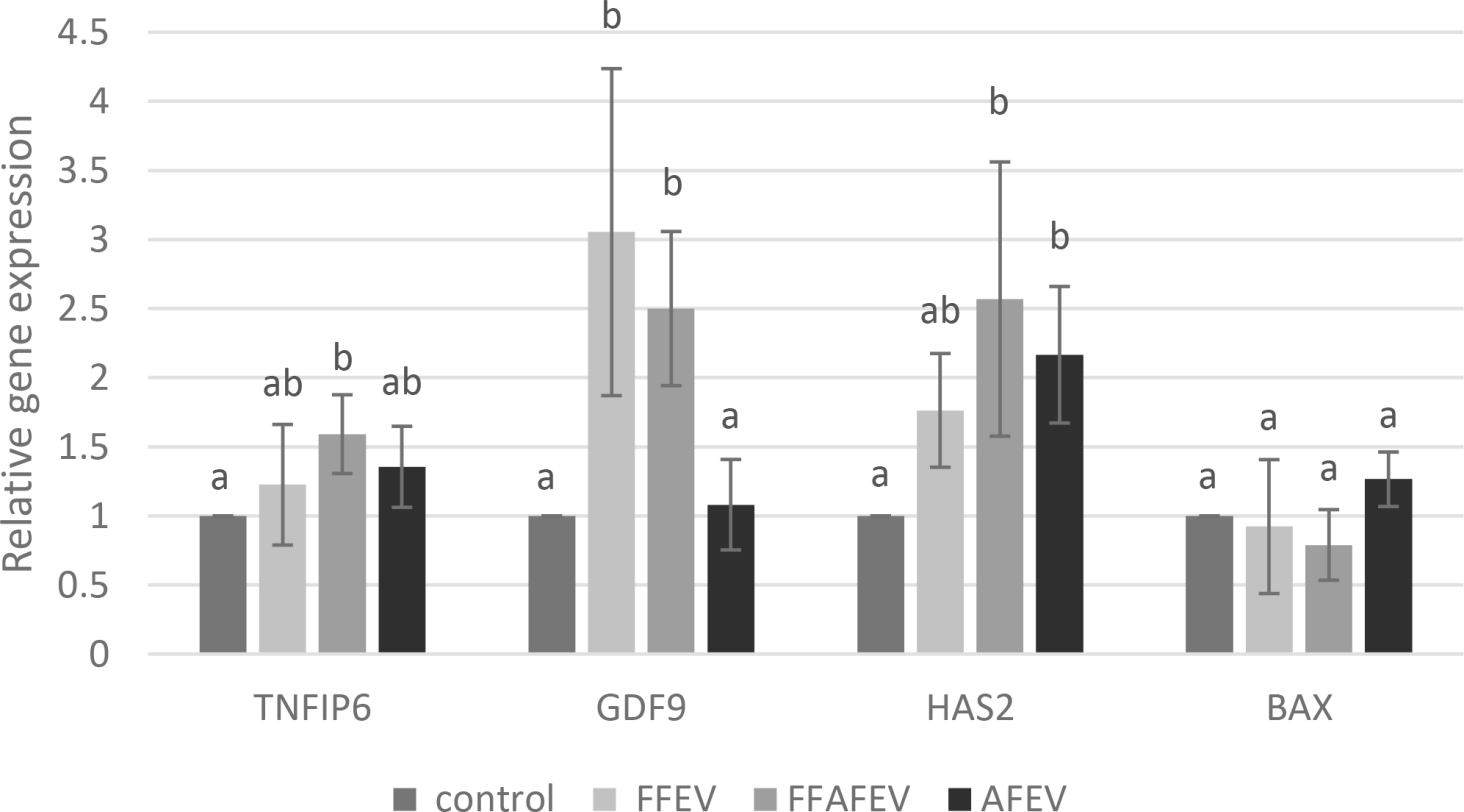
Effect of EVs on gene expression. Bars with different letters among groups are significantly different (P ≤ 0.05).

Quantitative real-time RT-PCR was performed to assess the expression of *GDF9, HAS2, TNFAIP6*, and *BAX* genes in the *in vitro* matured oocytes. The expression of *TNFAIP6* gene was significantly higher in the FFAFEV group than in the control group (p ≤ 0.05). Furthermore, the expression of the *HAS2* gene was significantly higher in the FFAFEV and AFEV groups than in the control group (p ≤ 0.05). The expression of the *GDF9* gene was significantly higher in the FFAFEV and FFEV groups than in the control and AFEV groups (p ≤ 0.05). No significant difference was found in the expression of the *BAX* gene among the groups (Fig 2).

#### Raman microspectroscopy analysis.

**Fig 3:**
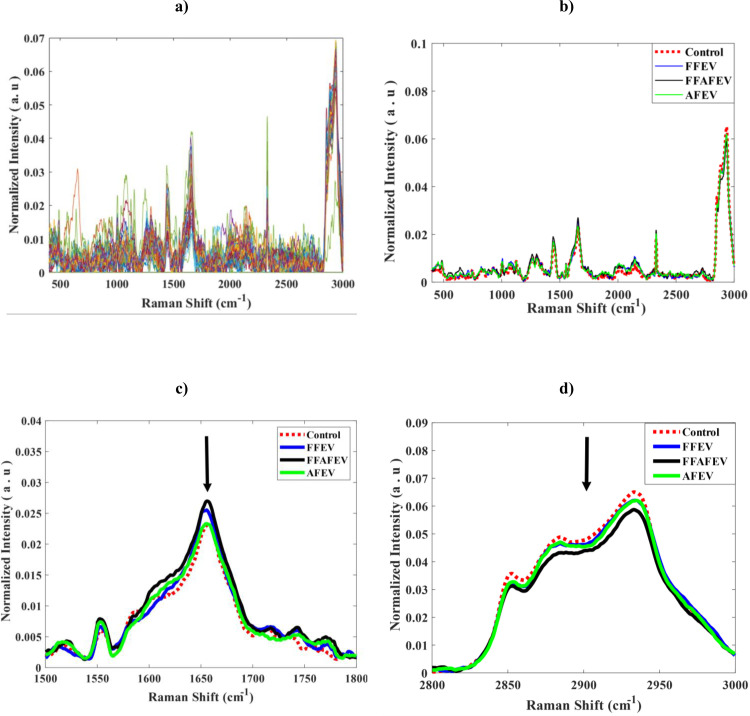
The Raman spectrum of the bovine oocytes: (a) the Raman spectrum of the bovine oocytes; (b) the average spectra recorded between 500 and 3000 cm^-1^; (c) a peak at 1655 cm^-1^ indicative of unsaturated lipids; and (d) a peak at 2833 cm^-1^ associated with saturated lipids.

**Fig 4:**
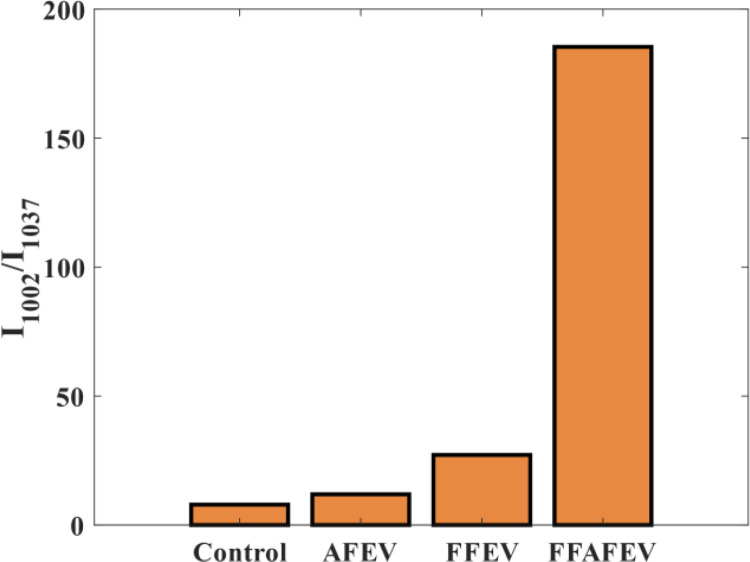
The ratio of phenylalanine at 1002 cm^-1^ to carbohydrate at 1037 cm^-1.^

As demonstrated in Fig 3a, the Raman spectrum of the bovine oocytes exhibited a complex profile characterized by a significant number of overlapping bands within the fingerprint region. The raw average spectra presented in Fig 3b, spanning the range of 500–3000 cm^-1,^ revealed distinct peaks at varying intensities across different regions. In Fig 3c, the 1602 cm^-1^ peak was observed, indicative of mitochondrial activity. Additionally, the peak at 1655 cm^-1^ that related to unsaturated lipids, in the FFAFEV and FFEV groups was higher than in the AFEV and control groups. In contrast, according to Fig 3d, the peak at 2883 cm^-1^ associated with saturated lipids, known to be detrimental to the oocyte development, exhibited a negative correlation with the control group, displaying a higher intensity than the other groups. In addition, the ratio of phenylalanine at 1002 cm^-1^ to carbohydrate at 1037 cm^-1^ in the FFAFEV group was greater than that of the control group (Fig 4).

**Fig 5:**
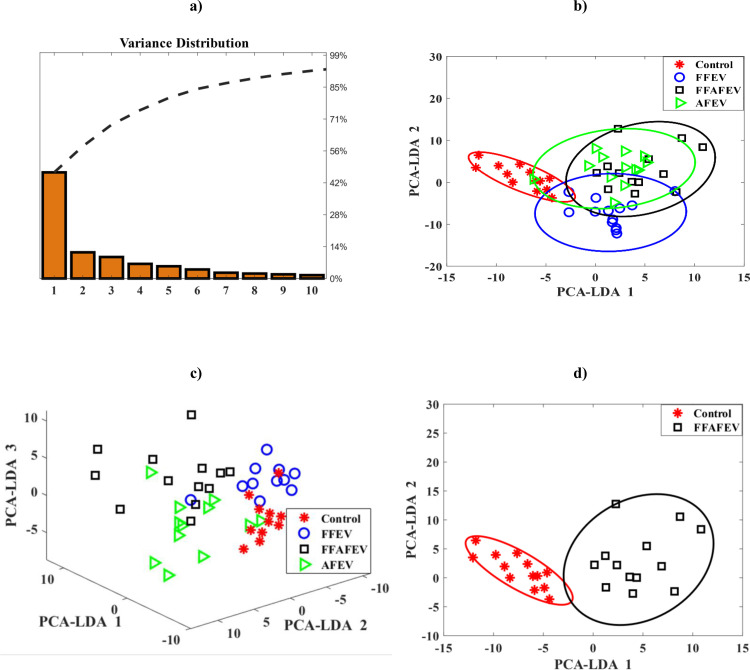
(a) Variance distribution plot from PCA, (b) and (c) two- and three-dimensional representation of the results of PCA-LDA conducted on the average spectra of four distinct groups, and (d) illustrates the PCA-LDA analysis comparing the FFAFEV group against the control group.

The PCA-LDA was performed to determine whether the effect of FFAFEV treatment was correctly classified according to specific Raman bands. Further, the data underwent to dimension reduction (Fig 5b and 5c) to have significant separation of the spectra. The variance distribution plot from PCA indicates that the first principal component accounts for most of the data variance, capturing the primary dispersion and group differences (Fig 5a). As Fig 5a shows, the sharp decrease in variance for the following components suggests that only a small number of components are sufficient to retain over 90% of the information, enabling effective dimensionality reduction while maintaining essential classification features. Fig 5d evidenced that spectra from the FFAFEV group were clearly distinguishable and significantly different from those of the control group, meanwhile, there was overlap among three EVs treatment groups (Fig 5b and 5c).

### Experiment 2

#### Effect of EVs on in vitro oocyte fertilization rate.

The detailed results of this study are presented in Table 3. The fertilization rate was significantly higher in the FFAFEV group than in the control and AFEV groups (p ≤ 0.05). Furthermore, the FFEV group exhibited a significantly higher fertilization rate than the AFEV group (p ≤ 0.05), although there was no significant difference between the FFEV group with the FFAFEV and control groups.

**Table 3:** Effect of experimental treatments on the *in vitro* fertilization (mean ± standard error).

Treatment	N	Normally fertilized n (%)	Non fertilized n (%)	Polyspermy n (%)
Control	117	72 (61.25 ± 2.88)^a^	40 (35.10 ± 3.00)	5 (3.63 ± 2.30)
FFEV	127	89 (70.57 ± 1.59)^ab^	34 (26.06 ± 1.61)	4 (3.35 ± 1.24)
FFAFEV	125	100 (80.07 ± 3.99)^b^	24 (19.25 ± 4.17)	1 (0.66 ± 0.66)
AFEV	128	70 (55.73 ± 2.28)^ac^	50 (37.11 ± 5.43)	8 (7.14 ± 4.26)

FFEV: cultured with Follicular fluid EVs; FFAFEV: cultured with Follicular fluid EVs and Ampulla fluid EVs; AFEV: cultured with Ampulla fluid EVs.

#### Effect of EVs on oocyte fertilization rate after ICSI.

Results of this study are shown in Table 4 in detail. There was no significant difference in the fertilization rate after ICSI among the groups (p ≥ 0.05).

**Table 4:** Effect of EVs treatments on the *in vitro* fertilization rate after ICSI (mean ± standard error).

Treatment	N	Fertilized after ICSI n (%)	Not fertilized after ICSI n (%)
Control	48	21 (44.69 ± 3.44)	27 (55.30 ± 3.44)
FFEV	52	26 (50.00 ± 4.50)	26 (50.00 ± 4.50)
FFAFEV	50	25 (50.00 ± 3.90)	25 (50.00 ± 3.90)
AFEV	48	21 (43.99 ± 1.74)	27 (56.00 ± 1.74)

FFEV: cultured with Follicular fluid EVs; FFAFEV: cultured with Follicular fluid EVs and Ampulla fluid EVs; AFEV: cultured with Ampulla fluid EVs.

## Discussion

The results of the present study showed that the supplementation of FF EVs during the initial 18 hours, along with AF EVs in the final 4.5 hours of *in vitro* maturation, increased both bovine oocyte maturation and fertilization rates. In support of our findings, Asaadi et al. showed that the follicular and ampullary fluid exosomes improved the *in vitro* blastocyst yield [17]. Lee et al. demonstrated that oviduct epithelial cell exosomes increased the *in vitro* maturation rate in canine [33]. Additionally, EVs derived from oviduct cells in porcine reduced polyspermy and increased active mitochondria resulting in increasing the efficiency of *in vitro* fertilization [34]. In contrast, Almiñana et al. observed that albeit oviductal EVs did not affect the fertilization rate however, the quality of the *in vitro* produced embryos was improved [35]. Moreover, isthmus oviductal exosomes had a positive effect on the cryotolerance of *in vitro* embryos [11]. The study by da Silveira et al. indicated that FF EVs contribute to optimizing *in vitro* embryo development [7]. Consequently, the use of follicular and AF EVs, which are involved in oocyte maturation, and embryo development, helps to simulate the physiological conditions in the laboratory and improve the quality of oocytes cultured *in vitro* [8,36]. In contrast to the favorable impact on fertilization rates following the addition of the EVs into the maturation medium in this study, we found no effects on fertilization rates when the oocytes were supplemented with EVs using the ICSI procedure. The interpretation of these results is challenging and necessitates further research with a larger sample size, however it is important to note that the fertilization rate was non-significantly higher in the FFAFEV oocytes. This may suggest that extending the culture of these fertilized oocytes could enhance the potential for increased cleavage and embryo development.

To explain the results of the present study, we investigated the gene expression related to oocyte and cumulus cell development to determine the quality of oocytes following treatment with follicular and AF EVs. Our findings revealed that the expression of the *TNFAIP6* gene in the FFAFEV group was significantly higher than the control group. Supporting this observation, Hung et al. observed that FF exosomes increase the expression of the *TNFAIP6* gene [36]. Furthermore, the expression of the *HAS2* gene in the current study was significantly higher in the FFAFEV and AFEV groups compared to the control group. Additionally, the expression of the *GDF9* gene in the FFEV group was significantly higher than in the control group. Lee et al. demonstrated that oviductal exosomes enhanced oocyte maturation and upregulated the expression of the *GDF9* and *TNFAIP6* genes in canine, although there was no significant difference in the *HAS2* gene [33]. In a study reported by Dalanezi et al., it was shown that FF EVs from the heat-stressed cows, compared to those from thermoneutral conditions increased expression of the *GDF9* and *HAS2* genes in cumulus cells [37]. In current study no significant difference were observed in the expression of the *BAX* gene, a marker of apoptosis. Lv et al. indicated that embryos cultured with exosomes showed reduced expression of the *BAX* gene in mice [38]. In contrast, Kusama et al. reported an increase in *BAX* gene expression in endometrial epithelial cells along with exosomes from 17 days pregnant cows. The rise in apoptotic gene expression was interpreted as evidence of the essential role of uterine fluid exosomes in placental implantation within the endometrial epithelial cells of the uterus [12].

In the present study, the quality of the oocytes following the addition of EVs was assessed using RMS. Our findings from the Raman spectral analysis showed that the peak at 1602 cm^-1^, associated with mitochondrial presence, was elevated in the FFAFEV and FFEV groups compared to the AFEV and control groups. Mitochondria are critical as the cellular energy source, and their re-distribution and abundance are vital for the cytoplasmic maturation of oocytes [39]. Previous studies have indicated that 1602 cm^-1^ peak was absent in fixed oocyte [21,27]. While in our study this peak was observed in fixed oocytes. In addition, we observed that the spectral range at 1655 cm^-1^, which corresponds to unsaturated lipids, was higher in the FFAFEV group than in the control group. Rusciano et al. demonstrated these lipids are a valuable energy source for oocytes [31]. Furthermore, Jimenez et al. identified the band 1656 cm^-1^, associated with beneficial cellular lipids. Additionally based on our findings, the band 2883 cm^-1^, related to detrimental saturated lipids for oocyte quality [40], showed a higher peak in the control group compared to the treatment groups. Jimenez et al. noted that during oocyte maturation, there is an increase in proteins and a decrease in carbohydrates, with the ratio of 1002/1037 serving as a marker for oocyte quality [40]. Our findings support this observation, as the ratio of phenylalanine at 1002 cm^-1^ to carbohydrate at 1037 cm^-1^ in the FFAFEV group was greater than that in the control group. PCA-LDA is particularly effective analysis for examining spectral variations among oocytes characterized by numerous variables per spectrum. This analytical approach transforms the data into a format that represents spectra as individual points in multivariate space. The PCA-LDA allows for grouping large multivariate data into different clusters by maximizing the inter-cluster separation and, at the same time, ensuring the minimum variability within the cluster [41]. In addition, PCA-LDA can effectively reduce the dimensionality of the spectral data, projecting it into a lower-dimensional space that highlights the greatest variance for optimal visualization. This approach enhances the separation of sample groups by preserving key discriminative features, thereby supporting precise classification and improved the interpretability of spectral patterns. The results of the PCA-LDA statistical analysis revealed a dispersion of data between the FFAFEV group and the control group, strongly suggesting that the FFAFEV treatment has a positive effect on the *in vitro* outcomes. Moreover, the application of EVs influenced the spectral characteristics and molecular fingerprint of the chemical structure of the oocyte.

## Conclusions

The results of the present study showed that supplementing EVs of follicular and ampullary fluid can be effectively employed for *in vitro* oocyte maturation. Additionally, the combination of EVs from follicular fluid during the initial 18 hours and ampullary fluid in the final 4.5 hours results in the maturation of the oocyte with a superior quality and higher fertilization rate when compared to the standard oocyte culture without EVs. Raman microspectroscopy results further indicate that the FFAFEV group has the highest number of mitochondria and unsaturated lipids, as well as the lowest ratio of saturated lipids. This powerful methodology has the potential to significantly enhance ART by providing in-depth insights into the health of the oocyte and its developmental potential.

## Supporting information

S1 Fig S1_raw_images. (PDF)

S1 File Supporting information. (RAR)
