# Supplementary material for: The effect of follicular and ampullary fluid extracellular vesicles on bovine oocyte competence and in vitro fertilization rates
Source: PLoS One. 2025 Jun 6;20(6):e0325268. doi: 10.1371/journal.pone.0325268 (PMC12143572; doi:10.1371/journal.pone.0325268)

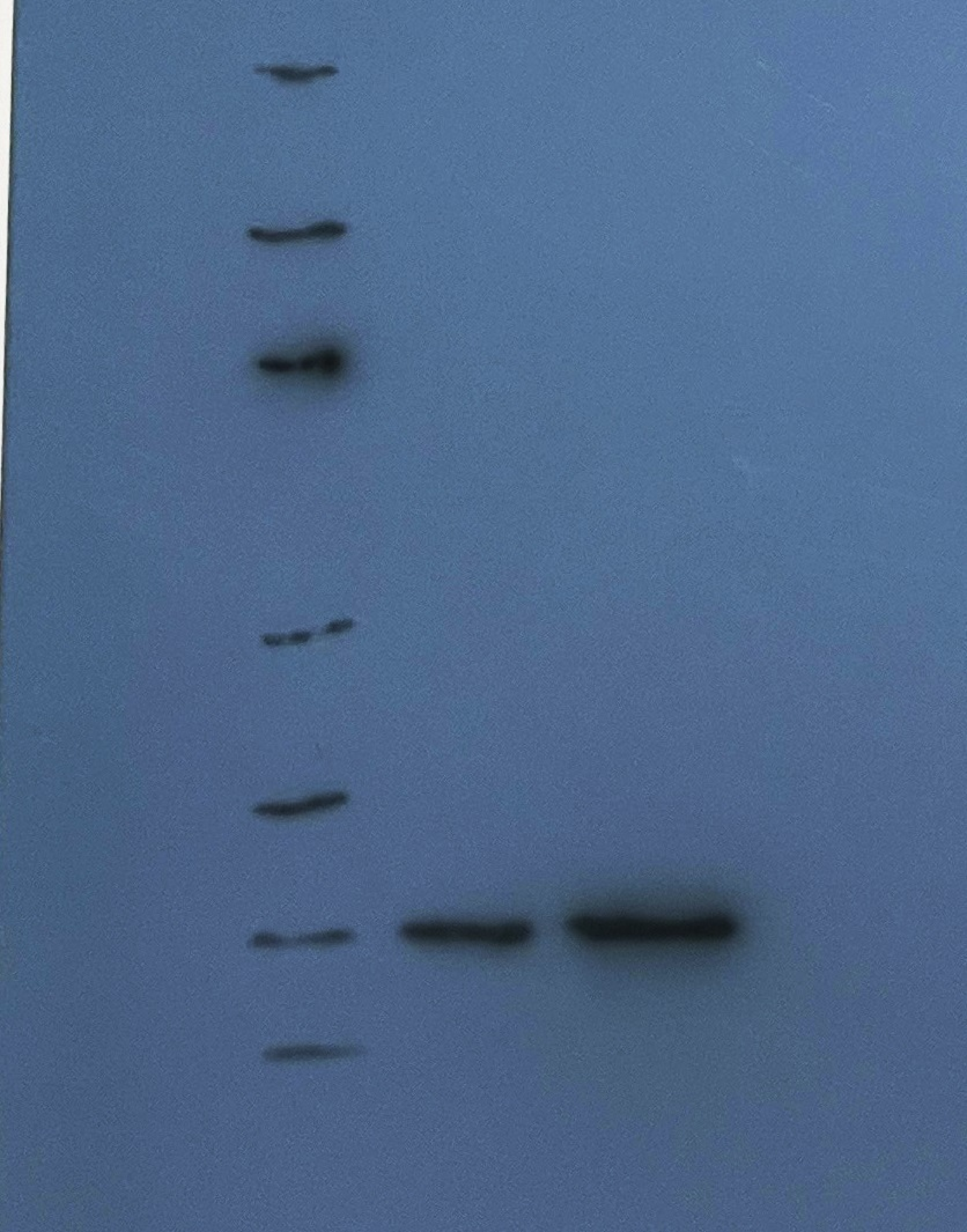

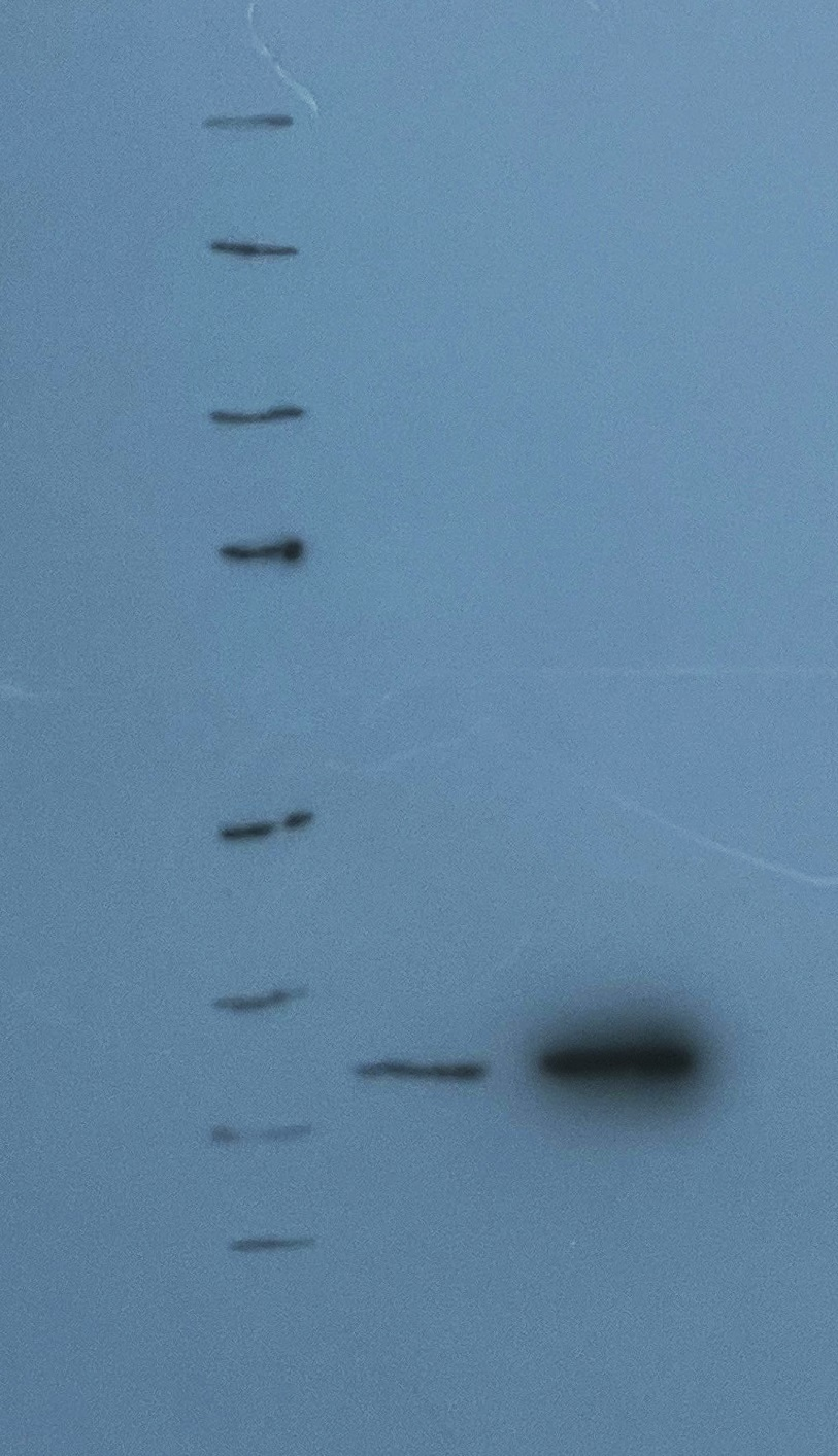

Supplement: S1 Fig — (PDF) [file pone.0325268.s001.pdf]
